# The saturation effect of body mass index on total lumbar bone mineral density for adults: The NHANES 2011–2020

**DOI:** 10.1097/MD.0000000000036838

**Published:** 2024-01-05

**Authors:** Cong Chen, Jun Jia, Peng Wang

**Affiliations:** a Department of Orthopedics, Weihai Municipal Hospital, Cheeloo College of Medicine, Shandong University, Shandong Province, P. R. China.

**Keywords:** body mass index, bone mineral density, lumbar, NHANES, osteoporosis

## Abstract

To explore the association between body mass index (BMI) and total lumbar bone mineral density (BMD) in adults. This study included 9927 participants from 2011 to 2020 National Health and Nutrition Examination Survey (NHANES). The date on BMI, total lumbar BMD and other covariates were collected. Multivariate linear regression analyses were performed to evaluate the association between BMI and total lumbar BMD. Smoothing curve fitting and saturation effects analysis models were used to analyze the nonlinear relationships and saturation values. Multivariate linear regression analyses revealed that BMI was positively linked to total lumbar BMD in non-adjusted models (β = 0.003, 95% CI: 0.003–0.003, *P* < .00001). After adjusting for gender and race (β = 0.003, 95% CI: 0.003–0.004, *P* < .00001) and all covariates (β = 0.004, 95% CI: 0.003–0.004, *P* < .00001), the association still existed. Smoothing curve fitting showed that there was nonlinear correlation between BMI and total lumbar BMD with saturation effect. The BMI saturation value was 21.2 kg/m^2^ in the total lumbar BMD based on saturation effects analysis models. There was nonlinear positive correlation between BMI and total lumbar BMD with saturation effect. For adults, keeping the BMI at a reasonable value (21.2 kg/m^2^) would obtain an optimal balance between BMI and total lumbar BMD.

## 1. Introduction

Osteoporosis is a common degenerative disease, characterized by weakened microarchitecture, reduced bone mineral density (BMD) and increased fragility.^[1–3]^ Osteoporosis can not only affect quality of life, but also increase healthcare burden.^[4]^ BMD is one of the important diagnostic indicators of osteoporosis.^[5,6]^

There are many factors related to osteoporosis, including gender, age, genetics, triglycerides, cholesterol, sociological factors and so on.^[7–10]^ In previous studies, body mass index (BMI) has been proven to be closely related to BMD.^[11–15]^ A study in a total of 10,910 participants over 50 years showed that BMI was positively related with BMD, and demonstrated keeping the BMI at a slightly overweight value (26 kg/m^2^) would obtain an optimal balance between BMI and BMD for people over 50 years old.^[11]^ In a cross-sectional study of 4056 adolescents, Wang et al indicated a positive saturation effect between BMI and BMD.^[12]^

To our knowledge, there are few studies on the relationship between BMI and BMD in adults. In the present study, we conducted a cross-sectional analysis based on the National Health and Nutrition Examination Survey (NHANES) database. The purpose of this study was to assessed the association between BMI and BMD in adults.

## 2. Materials and methods

### 2.1. Study population

**Figure 1.**
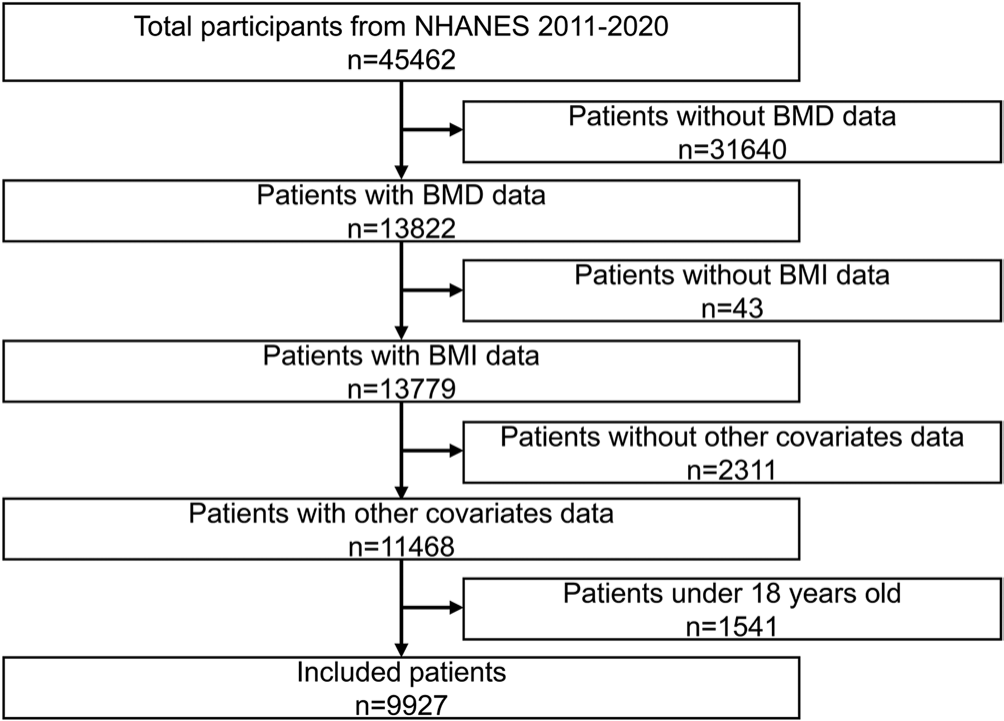
Flowchart of the patients.

The NHANES is a cross-sectional survey, which was authorized by the National Center for Health Statistics (NCHS) research ethics review board. And the informed consents were obtained from all participants. After excluding underlying diseases such as hyperthyroidism and diabetes, a total of 45,462 individuals were included initially from NHANES database (2011–2020). After excluded 31,640 individuals without BMD data, 43 individuals without BMI data, 2311 individuals without other covariates data, and 1541 individuals under 18 years old, leaving 9927 individuals enrolled in this study (Fig. 1).

### 2.2. Study variables

The independent variable was BMI, and the dependent variable was total lumbar BMD. Age, albumin (AL), alanine aminotransferase (ALT), aspartate transaminase (AST), alkaline phosphatase (ALP), blood urea nitrogen (BUN), calcium (CAL), creatine phosphokinase (CPK), bicarbonate (BC), creatinine (CR), gamma glutamyl transferase (GGT), glucose (GLU), iron (IR), phosphorus (PHO), bilirubin (BIL), total protein (PRO), uric acid (UA), sodium (SOD), potassium (POT), chloride (CL), osmolality (OSM), globulin (GLO), gender, race, and education level (EL) were all covariates. The detailed information on the measurement, calculation and interpretation of these variables can be obtained from the official NHANES website.

### 2.3. Statistical analyses

Multivariate regression analyses were performed to evaluate the association between BMI and total lumbar BMD. To characterize nonlinearity in the association between BMI and BMD, weighted generalized additive models and smooth curve fittings were further performed. Two-piece wise linear regression models were used to calculate the threshold effects if non-linearity associations existed. We employed 3 models: Model I, no adjustment for covariates; Model II, adjusted for gender, age and race; Model III, adjusted for all covariates. We used the software R (version 3.4.3) and Empower-Stats software (version 2.0, United States) for statistical analysis. The *P* < .05 was considered statistically significant.

## 3. Results

### 3.1. Baseline characteristics

A total of 9927 participants were included in the study. Among them, 4884 (49.2%) were males and 5043 (50.8%) were females. The average age of the patients was 46.173 ± 15.741 years. As shown in Table 1, the participants were divided into 4 subgroups based on BMI: underweight (< 18.5 kg/m^2^), normal (18.5–24.9 kg/m^2^), overweight (25–29.9 kg/m^2^), and obese (≥ 30 kg/m^2^). There were significant differences in all covariates among the BMI category, including age, AL, ALT, AST, ALP, BUN, CAL, CPK, BC, CR, GGT, GLU, IR, PHO, BIL, PRO, UA, SOD, POT, CL, OSM, GLO, gender, race, and EL. With increased BMI, the total lumbar BMD significantly increased (*P* < .00001), and the highest total lumbar BMD was in the Q4 group. As shown in Table 2, the participants were divided into 4 subgroups based gender. There were significant differences in most covariates except ALP.

**Table 1:** Weighted characteristics of study population based on BMI quartiles.

BMI (kg/m^2^)	Q1 (< 18.5)	Q2 (18.5–24.9)	Q3 (25–29.9)	Q4 (≥ 30)	*P* value
Age (yr)	34.488 ± 16.511	42.151 ± 15.599	46.033 ± 14.181	45.826 ± 13.566	<0.00001
AL (g/L)	45.030 ± 3.443	44.192 ± 3.314	43.583 ± 3.187	42.132 ± 3.325	<0.00001
ALT (U/L)	18.899 ± 11.301	21.462 ± 16.136	25.269 ± 15.804	29.500 ± 20.224	<0.00001
AST (U/L)	24.210 ± 16.129	24.488 ± 14.353	24.784 ± 10.740	26.075 ± 16.462	0.00002
ALP (IU/L)	65.690 ± 23.195	62.858 ± 21.558	67.706 ± 21.318	71.759 ± 24.589	<0.00001
BUN (mmol/L)	4.095 ± 1.892	4.664 ± 1.631	4.899 ± 1.608	4.772 ± 1.804	<0.00001
CAL (mmol/L)	2.359 ± 0.081	2.355 ± 0.082	2.348 ± 0.084	2.333 ± 0.088	<0.00001
CPK (IU/L)	107.338 ± 75.645	137.893 ± 174.248	157.900 ± 201.394	155.678 ± 147.436	<0.00001
BC (mmol/L)	24.965 ± 2.536	25.331 ± 2.116	25.081 ± 2.172	24.452 ± 2.306	<0.00001
CR (umol/L)	71.769 ± 38.753	75.207 ± 29.318	78.270 ± 24.016	76.497 ± 27.620	0.00002
GGT (IU/L)	22.740 ± 51.646	22.475 ± 45.386	27.852 ± 35.836	33.199 ± 42.283	<0.00001
GLU (mmol/L)	4.877 ± 0.946	5.120 ± 1.520	5.416 ± 1.701	5.893 ± 2.253	<0.00001
IR (umol/L)	16.007 ± 6.490	16.696 ± 6.839	16.165 ± 6.388	14.265 ± 5.984	<0.00001
PHO (mmol/L)	1.261 ± 0.184	1.229 ± 0.177	1.192 ± 0.175	1.185 ± 0.177	<0.00001
BIL (umol/L)	12.075 ± 6.774	11.443 ± 5.478	11.027 ± 5.241	9.721 ± 5.048	<0.00001
PRO (g/L)	72.920 ± 4.609	71.437 ± 4.790	71.090 ± 4.341	70.909 ± 4.385	<0.00001
UA (mg/dL)	4.382 ± 1.198	4.842 ± 1.170	5.420 ± 1.308	5.758 ± 1.381	<0.00001
SOD (mmol/L)	139.358 ± 2.148	139.162 ± 2.158	139.199 ± 2.181	139.055 ± 2.347	0.02583
POT (mmol/L)	3.878 ± 0.384	3.955 ± 0.335	3.966 ± 0.332	3.985 ± 0.324	0.00001
CL (mmol/L)	103.356 ± 2.730	103.224 ± 2.623	103.318 ± 2.799	103.628 ± 2.982	<0.00001
OSM (mmol/kg)	277.049 ± 4.609	277.462 ± 4.820	278.053 ± 4.603	278.137 ± 5.084	<0.00001
GLO (g/L)	27.890 ± 4.095	27.245 ± 4.254	27.506 ± 4.165	28.777 ± 4.263	<0.00001
Gender (%)					<0.00001
Male	37.895	46.170	56.819	47.922	-
Female	62.105	53.830	43.181	52.078	-
Race (%)					<0.00001
Non-Hispanic White	58.987	65.189	64.935	61.419	-
Non-Hispanic Black	13.521	8.955	9.573	15.008	-
Mexican American	4.413	5.919	9.955	11.460	-
Other race	23.079	19.937	15.537	12.114	-
EL (%)					<0.00001
Less than HS	32.380	28.906	34.981	37.320	-
HS	52.438	66.358	63.197	60.968	-
More than HS	15.182	4.736	1.822	1.713	-
Total lumbar BMD (g/cm^2^)	0.94 ± 0.15	1.01 ± 0.15	1.03 ± 0.15	1.05 ± 0.15	<0.00001

Mean ± SD for continuous variables: the *P* value was calculated by the weighted linear regression model. % for categorical variables: the *P* value was calculated by the weighted chi-square test.

AL = albumin, ALP = alkaline phosphatase, ALT = alanine aminotransferase, AST = aspartate transaminase, BC = bicarbonate, BIL = bilirubin, BMD = bone mineral density, BMI = body mass index, BUN = blood urea nitrogen, CAL = calcium, CL = chloride, CPK = creatine phosphokinase, CR = creatinine, EL = education level, GGT = gamma glutamyl transferase, GLO = globulin, GLU = glucose, HS = high school, IR = iron, OSM = osmolality, PHO = phosphorus, POT = potassium, PRO = total protein, SOD = sodium, UA = uric acid.

**Table 2:** Weighted characteristics of study population based on gender.

Gender	Male	Female	*P* value
Age (yr)	43.482 ± 14.284	45.886 ± 14.756	<0.00001
AL (g/L)	44.262 ± 3.324	42.207 ± 3.152	<0.00001
ALT (U/L)	30.125 ± 20.071	21.166 ± 14.101	<0.00001
AST (U/L)	27.118 ± 13.359	23.208 ± 14.741	<0.00001
ALP (IU/L)	67.398 ± 21.812	68.215 ± 24.059	0.07625
BUN (mmol/L)	5.028 ± 1.652	4.517 ± 1.707	<0.00001
CAL (mmol/L)	2.353 ± 0.084	2.336 ± 0.086	<0.00001
CPK (IU/L)	195.159 ± 208.486	105.735 ± 113.580	<0.00001
BC (mmol/L)	25.192 ± 2.178	24.639 ± 2.275	<0.00001
CR (umol/L)	86.106 ± 28.511	67.104 ± 22.121	<0.00001
GGT (IU/L)	32.145 ± 37.940	24.292 ± 44.668	<0.00001
GLU (mmol/L)	5.588 ± 1.983	5.413 ± 1.811	<0.00001
IR (umol/L)	17.066 ± 6.380	14.137 ± 6.208	<0.00001
PHO (mmol/L)	1.181 ± 0.183	1.221 ± 0.169	<0.00001
BIL (umol/L)	12.025 ± 5.841	9.315 ± 4.330	<0.00001
PRO (g/L)	71.608 ± 4.485	70.686 ± 4.474	<0.00001
UA (mg/dL)	6.000 ± 1.225	4.726 ± 1.163	<0.00001
SOD (mmol/L)	139.218 ± 2.148	139.055 ± 2.322	0.00028
POT (mmol/L)	4.014 ± 0.329	3.922 ± 0.327	<0.00001
CL (mmol/L)	103.135 ± 2.763	103.682 ± 2.861	<0.00001
OSM (mmol/kg)	278.385 ± 4.563	277.414 ± 5.091	<0.00001
GLO (g/L)	27.346 ± 4.092	28.478 ± 4.388	<0.00001
RACE			0.00146
Non-Hispanic White	64.123	63.095	-
Non-Hispanic Black	10.34	12.623	-
Mexican American	9.876	8.688	-
Other race	15.662	15.595	-
EL (%)			<0.00001
Less than high school	36.677	31.473	-
High school	59.923	66.31	-
More than high school	3.4	2.217	-
Total lumbar BMD (g/cm^2^)	1.039 ± 0.154	1.020 ± 0.151	<0.00001
BMI	28.734 ± 5.962	29.234 ± 7.308	0.00019

Mean ± SD for continuous variables: the *P* value was calculated by the weighted linear regression model. % for categorical variables: the *P* value was calculated by the weighted chi-square test.

AL = albumin, ALP = alkaline phosphatase, ALT = alanine aminotransferase, AST = aspartate transaminase, BC = bicarbonate, BIL = bilirubin, BMD = bone mineral density, BMI = body mass index, BUN = blood urea nitrogen, CAL = calcium, CL = chloride, CPK = creatine phosphokinase, CR = creatinine, EL = education level, GGT = gamma glutamyl transferase, GLO = globulin, GLU = glucose, IR = iron, OSM = osmolality, PHO = phosphorus, POT = potassium, PRO = total protein, SOD = sodium, UA = uric acid.

### 3.2. Relationship between BMI and total lumbar BMD

The results of multivariate linear regression analyses were shown in Table 3. In all models, BMI was strongly positively associated with total lumbar BMD: model I (β = 0.003, 95% CI: 0.003–0.003, *P* < .00001); model II (β = 0.003, 95% CI: 0.003–0.004, *P* < .00001); and model III (β = 0.004, 95% CI: 0.003–0.004, *P* < .00001). Moreover, when the BMI was categorized for analysis, the trend remained significant (p for trend < 0.001). In a subgroup analysis stratified by gender after adjusting for all covariates, a positive association between BMI and total lumbar BMD was also found in both men (β = 0.004, 95% CI: 0.003–0.005, *P* < .00001) and women (β = 0.004, 95% CI: 0.004–0.005, *P* < .00001). In a subgroup analysis stratified by age after adjusting for all covariates, a positive association between BMI and total lumbar BMD still existed among the different age groups (young group, β = 0.001, 95% CI: 0.000–0.001, *P* = .01265; middle-aged group, β = 0.005, 95% CI: 0.005–0.005, *P* < .00001; old group, β = 0.013, 95% CI: 0.011–0.016, *P* < .00001). In a subgroup analysis stratified by race after adjusting for all covariates, the positive associations between BMI and total lumbar BMD remained significant in whites (β = 0.004, 95% CI: 0.003–0.005, *P* < .00001), blacks (β = 0.004, 95% CI: 0.003–0.005, *P* < .00001), Mexicans (β = 0.003, 95% CI: 0.002–0.004, *P* < .00001), and other races (β = 0.004, 95% CI: 0.003–0.004, *P* < .00001).

**Table 3:** Association between BMI and total lumbar BMD.

	Model I β (95% CI) *P* value	Model II β (95% CI) *P* value	Model III β (95% CI) *P* value
BMI (kg/m^2^)	0.003 (0.003, 0.003) < 0.00001	0.003 (0.003, 0.004) < 0.00001	0.004 (0.003, 0.004) < 0.00001
Q1 (< 18.5)	Reference	Reference	Reference
Q2 (18.5–24.9)	0.075 (0.050, 0.100) < 0.00001	0.089 (0.065, 0.113) < 0.00001	0.084 (0.060, 0.108) < 0.00001
Q3 (25–29.9)	0.088 (0.063, 0.113) < 0.00001	0.107 (0.083, 0.131) < 0.00001	0.106 (0.082, 0.130) < 0.00001
Q4 (≥ 30)	0.108 (0.083, 0.133) < 0.00001	0.125 (0.101, 0.149) < 0.00001	0.128 (0.104, 0.153) < 0.00001
P for trend	< 0.001	< 0.001	< 0.001
Subgroup analysis stratified by gender			
Male	0.003 (0.002, 0.004) < 0.00001	0.003 (0.002, 0.004) < 0.00001	0.003 (0.002, 0.004) < 0.00001
Female	0.003 (0.003, 0.004) < 0.00001	0.003 (0.003, 0.004) < 0.00001	0.003 (0.003, 0.004) < 0.00001
Subgroup analysis stratified by age			
Young (18–44)	0.001 (0.000, 0.001) 0.01265	0.001 (0.000, 0.001) 0.01265	0.001 (0.000, 0.001) 0.01265
Middle-aged (45–69)	0.005 (0.005, 0.006) < 0.00001	0.005 (0.005, 0.006) < 0.00001	0.005 (0.005, 0.006) < 0.00001
Old (>=70)	0.013 (0.011, 0.016) < 0.00001	0.013 (0.011, 0.016) < 0.00001	0.013 (0.011, 0.016) < 0.00001
Subgroup analysis stratified by race			
Non-Hispanic White	0.003 (0.002, 0.004) < 0.00001	0.003 (0.002, 0.004) < 0.00001	0.003 (0.002, 0.004) < 0.00001
Non- Hispanic Black	0.003 (0.002, 0.004) < 0.00001	0.003 (0.002, 0.004) < 0.00001	0.003 (0.002, 0.004) < 0.00001
Mexican American	0.002 (0.001, 0.003) 0.00021	0.002 (0.001, 0.003) 0.00021	0.002 (0.001, 0.003) 0.00021
Other race	0.003 (0.002, 0.004) < 0.00001	0.003 (0.002, 0.004) < 0.00001	0.003 (0.002, 0.004) < 0.00001

Model I, no adjustment for covariates; Model II, adjusted for gender, age and race; Model III, adjusted for all covariates.

BMI = body mass index.

### 3.3. Saturating effect for BMI and total lumbar BMD

**Figure 2.**
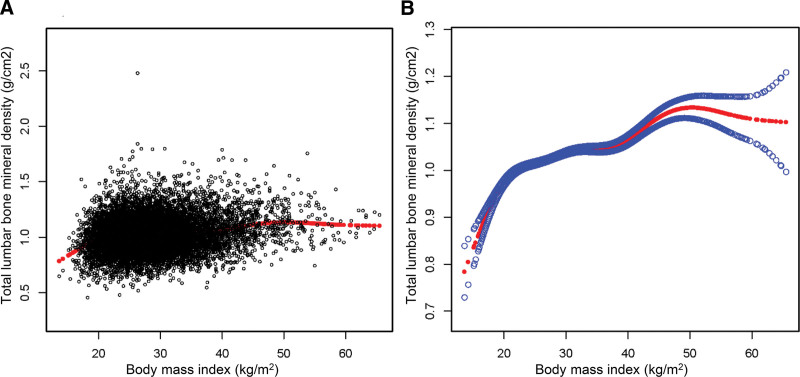
The association between BMI and total lumbar bone BMD. (A) Each black point represents a sample. (B) The solid red line represents the smooth curve fit between variables. Blue bands represent the 95% confidence interval from the fit. Age, AL, ALT, AST, ALP, BUN, CAL, CPK, BC, CR, GGT, GLU, IR, PHO, BIL, PRO, UA, SOD, POT, CL, OSM, GLO, gender, race, and EL were adjusted. AL = albumin, ALP = alkaline phosphatase, ALT = alanine aminotransferase, AST = aspartate transaminase, BC = bicarbonate, BIL = bilirubin, BUN = blood urea nitrogen, CAL = calcium, CL = chloride, CPK = creatine phosphokinase, CR = creatinine, GGT = gamma glutamyl transferase, GLO = globulin, GLU = glucose, IR = iron, OSM = osmolality, PHO = phosphorus, POT = potassium, PRO = total protein, SOD = sodium.

Smoothing curve fitting showed that there was nonlinear correlation between BMI and total lumbar BMD with saturation effect (Fig. 2). The BMI saturation value was 21.2 kg/m^2^ in the total lumbar BMD based on saturation effects analysis models (Table 4). When BMI < 21.2 kg/m^2^, the total lumbar BMD increased by 0.024 g/cm^2^ per unit rise in BMI (95% CI: 0.019–0.030). However, for each unit rise in BMI > 21.2 kg/m^2^, the total lumbar BMD rose only by 0.003 kg/m^2^ (95% CI: 0.003–0.004). When the BMI exceeded saturation value, the total lumbar BMD rose very slowly.

**Table 4:** Saturation effect analysis of BMI on total lumbar BMD.

Total lumbar BMD	Adjusted β (95% CI) P
Fitting by the standard linear model	0.004 (0.003, 0.004) < 0.0001
Fitting by 2-piecewise linear model	
Inflection point (K), kg/m^2^	21.2
< K, effect 1	0.024 (0.019, 0.030) < 0.0001
> K, effect 2	0.003 (0.003, 0.004) < 0.0001
Effect 2-1	-0.021 (-0.027, -0.016) < 0.0001
Log likelihood ratio	< 0.001

BMD = bone mineral density, BMI = body mass index.

## 4. Discussion

### 4.1. Relationship between BMI and BMD

There are many factors related to osteoporosis, including gender, age, genetics, triglycerides, cholesterol, sociological factors and so on.^[7–10]^ In previous studies, body mass index (BMI) has been proven to be closely related to BMD.^[11–15]^ In a cross-sectional study of 4056 adolescents ages 12 to 19, Wang et al found that BMI was significantly associated with total BMD, which remained significant in subgroups stratified by gender, age, ethnicity, and standing height.^[12]^ Li et al evaluated 2218 participants aged 40 to 59 years, and found that BMI was positively related with lumbar BMD in middle-aged adults.^[13]^ Another study based on 3296 participants aged 50 years and older confirmed a positive correlation between BMI and BMD in older adults.^[14]^ And results of a cross-sectional study of 10,910 participants over 50 years revealed that BMI was positively associated with lumbar spine BMD and femoral BMD.^[11]^ In this study, we found that BMI was strongly positively associated with total lumbar BMD. Moreover, when the BMI was categorized for analysis, the trend remained significant (p for trend < 0.001). And in the subgroup analysis stratified by gender, age and race, the positive associations between BMI and total lumbar BMD remained significant. However, the bias caused by other residual confounding factors, such as sex hormones, may exist. In our supplementary analysis from NHANES database (2013–2016), we found estrogen was an independent risk factor affecting BMD (Supplement-Table 1, http://links.lww.com/MD/L276), but the inclusion of estrogen levels as covariates did not alter the conclusions drawn in the present study (Supplement-Table 2, http://links.lww.com/MD/L277).

### 4.2. Saturating effect for BMI and BMD

In a cross-sectional study of 4056 adolescents ages 12 to 19, Wang et al indicated a positive saturation effect between BMI and BMD, and the saturation effect values of BMI was 22 kg/m^2^.^[12]^ Li et al evaluated 2218 participants aged 40 to 59 years, and found an inverted U-shaped association between BMI and BMD, with a saturation value at approximately 50 kg/m^2^.^[13]^ Another study based on 10,910 participants aged 50 years and older confirmed that there was a saturation value association between BMI and BMD, and the saturation effect values of BMI was around 26 kg/m^2^.^[11]^ And results of a cross-sectional study of 2903 participants over 50 years revealed that a saturation value between BMI and BMD, and the BMI saturation value was 24.3 kg/m^2^.^[15]^ In the present study, we observed a nonlinear association between BMI and total lumbar BMD, with a point of inflection at 21.2 kg/m^2^. This may imply that a BMI below 21.2 kg/m^2^ has independent protective effects on bone health, whereas a BMI exceeding 21.2 kg/m^2^ weakens this protective effect.

### 4.3. Limitations

Firstly, this study was a cross-sectional study, which cannot determine the causal relationship between BMI and BMD. Secondly, database limitations may lead to data bias, and affect the result. Third, the bias caused by residual confounding factors remains.

## 5. Conclusion

There was nonlinear positive correlation between BMI and total lumbar BMD with saturation effect. For adults over 18 years old, keeping the BMI at a reasonable value (21.2 kg/m^2^) would obtain an optimal balance between BMI and total lumbar BMD.

## Author contributions

**Conceptualization:** Peng Wang.

Data curation: Jun Jia.

Formal analysis: Cong Chen.

Funding acquisition: Peng Wang.

Investigation: Cong Chen.

Methodology: Cong Chen.

Software: Cong Chen.

Supervision: Peng Wang.

Validation: Peng Wang.

Visualization: Peng Wang.

Writing – original draft: Cong Chen.

Writing – review & editing: Peng Wang.

## Supplementary Material
