# Supplementary material for: The saturation effect of body mass index on total lumbar bone mineral density for adults: The NHANES 2011–2020
Source: Medicine (Baltimore). 2024 Jan 5;103(1):e36838. doi: 10.1097/MD.0000000000036838 (PMC10766303; doi:10.1097/MD.0000000000036838)
Supplement: Supplementary file 1 [file medi-103-e36838-s001.docx]

**Supplement-Table 1 Association between Sex Steroid Hormone (Testosterone, Sex Hormone Binding Globulin, and Estradiol) and total lumbar BMD**

|  | Model I  OR (95% CI) P | Model II  OR (95% CI) P | Model III  OR (95% CI) P |
| --- | --- | --- | --- |
| Testosterone | 0.000 (-0.000, 0.000) 0.77838 | 0.000 (0.000, 0.000) 0.00932 | 0.000 (-0.000, 0.000) 0.07486 |
| SHBG | 0.000 (0.000, 0.000) 0.01076 | 0.000 (-0.000, 0.000) 0.29262 | 0.000 (-0.000, 0.000) 0.63486 |
| Estradiol | 0.000 (0.000, 0.000) <0.00001 | 0.000 (0.000, 0.000) <0.00001 | 0.000 (0.000, 0.000) <0.00001 |

Model I, no adjustment for covariates; Model II, adjusted for gender, age and race; Model III, adjusted for all covariates, SHBG, Sex Hormone Binding Globulin.
