# Supplementary material for: The saturation effect of body mass index on total lumbar bone mineral density for adults: The NHANES 2011–2020
Source: Medicine (Baltimore). 2024 Jan 5;103(1):e36838. doi: 10.1097/MD.0000000000036838 (PMC10766303; doi:10.1097/MD.0000000000036838)
Supplement: Supplementary file 2 [file medi-103-e36838-s002.docx]

Supplement-Table 2 Association between estradiol and total lumbar BMD

|  | Model I  OR (95% CI) P | Model II  OR (95% CI) P | Model III  OR (95% CI) P | Model IV  OR (95% CI) P |
| --- | --- | --- | --- | --- |
| BMI | 0.001 (0.000, 0.001) 0.00207 | 0.001 (0.000, 0.001) 0.00114 | 0.001 (0.000, 0.001) 0.00052 | 0.002 (0.001, 0.003) <0.00001 |
| Stratified by BMI（kg/m^2^） | | | | |
| <18.5 | Reference | Reference | Reference | Reference |
| 18.5-24.9 | 0.060 (0.029, 0.091) 0.00015 | 0.069 (0.039, 0.099) <0.00001 | 0.068 (0.038, 0.098) 0.00001 | 0.070 (0.040, 0.100) <0.00001 |
| 25-29.9 | 0.060 (0.029, 0.091) 0.00016 | 0.076 (0.046, 0.106) <0.00001 | 0.075 (0.045, 0.105) <0.00001 | 0.083 (0.052, 0.113) <0.00001 |
| >=30 | 0.056 (0.025, 0.087) 0.00038 | 0.069 (0.038, 0.099) <0.00001 | 0.068 (0.038, 0.098) <0.00001 | 0.080 (0.049, 0.112) <0.00001 |
| Stratified by sex | | | | |
| Male | 0.001 (0.000, 0.002) 0.01309 | 0.001 (0.000, 0.002) 0.00394 | 0.001 (-0.000, 0.002) 0.15863 | 0.002 (0.001, 0.003) 0.00045 |
| Female | 0.001 (-0.000, 0.001) 0.07368 | 0.001 (0.000, 0.001) 0.01823 | 0.001 (0.000, 0.002) 0.00688 | 0.001 (0.001, 0.002) 0.00081 |
| Stratified by age (year) | | | | |
| 18-44 | 0.000 (-0.001, 0.001) 0.74777 | -0.000 (-0.001, 0.000) 0.56359 | -0.000 (-0.001, 0.000) 0.61395 | 0.002 (0.001, 0.003) 0.00001 |
| 45-69 | 0.003 (0.002, 0.004) <0.00001 | 0.003 (0.002, 0.004) <0.00001 | 0.003 (0.002, 0.004) <0.00001 | 0.003 (0.002, 0.004) <0.00001 |
| Stratified by race | | | | |
| White | 0.001 (-0.000, 0.002) 0.14108 | 0.001 (-0.000, 0.002) 0.05357 | 0.001 (0.000, 0.002) 0.03507 | 0.002 (0.001, 0.003) 0.00044 |
| Black | 0.000 (-0.001, 0.001) 0.69243 | 0.001 (-0.000, 0.002) 0.06991 | 0.001 (-0.000, 0.002) 0.09154 | 0.001 (-0.001, 0.002) 0.29345 |
| Mexican | 0.000 (-0.001, 0.002) 0.52010 | 0.001 (-0.000, 0.002) 0.14285 | 0.001 (-0.000, 0.002) 0.08716 | 0.001 (-0.000, 0.003) 0.08657 |
| Other | 0.001 (0.000, 0.002) 0.03832 | 0.001 (0.000, 0.002) 0.02498 | 0.001 (0.000, 0.003) 0.00829 | 0.003 (0.001, 0.004) 0.00006 |

Model I, no adjustment for covariates; Model II, adjusted for gender, age and race; Model III, adjusted for adjusted for gender, age, race and estradiol; Model IV, adjusted for all covariates.

Data missed for individuals over 70 years old on subgroups analysis stratified by age.
